# Enclosure enhances grassland plant diversity and community stability more effectively than grazing and mowing by strengthening species turnover regulation and soil process driving

**DOI:** 10.3389/fpls.2026.1747083

**Published:** 2026-02-18

**Authors:** Mengyuan Shi, Tianqi Yu, Sisi Chen, Chu Zhang, Xiaoping Xin, Senya He, Fanglin Liu, Li Ji, Ying Li, Xiaotian Gao, Ruirui Yan

**Affiliations:** 1 State Key Laboratory of Efficient Utilization of Arable Land in China, Institute of Agricultural Resources and Regional Planning, Chinese Academy of Agricultural Sciences, Beijing, China; 2 College of Grassland Science, Xinjiang Agricultural University, Urumqi, China

**Keywords:** community stability, enclosure, environmental factor drivers, grazing, mowing, plant diversity, temperate meadow steppe

## Abstract

Grassland ecosystems are profoundly influenced by land management practices, yet the long-term mechanisms linking plant diversity and community stability remain unclear. In this study, we conducted three-year observational study to assess how enclosure, grazing, and mowing affect plant community dynamics through species turnover, niche overlap, and environmental drivers in the Hulunbuir temperate meadow steppe of Inner Mongolia. Using 198 permanent quadrats monitored over three consecutive growing seasons, we quantified α-, β-, and γ-diversity and assessed stability via biomass variability. Enclosure was associated with higher species richness and lower biomass variability, suggesting enhanced community stability under reduced disturbance, whereas grazing was associated with lower species richness and greater temporal and spatial variability in community structure, potentially linked to intensified species turnover and soil compaction. Mowing generally showed intermediate patterns, reflecting moderate alteration of competitive dynamics and community composition. Our analyses further revealed that soil physical properties and nutrient availability—particularly soil bulk density(SBD), total nitrogen (TN), and organic carbon (OC)—were key environmental factors associated with variation in plant diversity and stability across management regimes. Structural equation modelling based on observational data indicated that these environmental factors may influence diversity and stability both directly and indirectly, with pathways differing among management types. Our findings indicate that grassland management practices modulate diversity–stability relationships in a management-dependent manner, likely through their effects on species turnover, niche structure, and soil–plant feedbacks. These results highlight the importance of context-specific management strategies for sustaining grassland stability under ongoing environmental change. temperate meadow steppe; plant diversity; community stability; enclosure; grazing; mowing; environmental factor drivers

## 1 Introduction

Species diversity and community stability are among the most fundamental concepts in ecology, and they involve complex interactions (Bezemer and van der Putten, 2007). Not only do they reflect ecosystem health, but they also play crucial roles in maintaining the ecological balance, species persistence, and provision of ecosystem services (Mougi, 2024). Studies have shown that high-diversity plant communities can increase ecosystem resistance to environmental disturbances by increasing functional redundancy among species, thereby improving community stability (Naeem et al., 1994). However, the strength and direction of diversity–stability relationships are often context dependent and may vary with disturbance regimes and management practices, particularly in grassland ecosystems. Therefore, gaining a deeper understanding of the relationship between plant diversity and community stability under contrasting land management practices has significant ecological and management implications.

Grassland ecosystems, as one of the most important biomes globally, are closely linked to key ecological services, such as grassland productivity, carbon storage, and soil protection, through their plant diversity and community stability (Tilman, 1996; Loreau et al., 2001). The impact of land management practices on grassland plant communities is a key issue in grassland ecology research (Drose et al., 2021; Zhu et al., 2024). Different land management strategies, notably enclosure, grazing, and mowing, not only directly interfere with species composition but also may influence community stability by altering the dynamic processes of resource competition, species turnover, and niche overlap (Milchunas and Lauenroth, 1993; Xu et al., 2010). Enclosure can reduce or eliminate the negative effects of grazing, promote plant recovery and increase diversity (Schultz et al., 2011). Grazing, on the other hand, affects plant population dynamics through selective feeding by herbivores, often leading to a reduction in dominant species and a decline in species richness (Gao and Carmel, 2020). Mowing alters the structure and productivity of plant communities, potentially imposing significant effects on species survival strategies and community stability (Weiner et al., 2011). Although these three management practices are widely applied, their ecological effects are not directly comparable because they differ fundamentally in disturbance type, intensity, and temporal frequency. Many studies have shown that plant diversity is one of the key factors maintaining the stability and functionality of grassland ecosystems (Tilman, 1996; Yang et al., 2011). Specifically, under different land management practices, changes in plant diversity have profound impacts on grassland ecosystem productivity, carbon storage, and hydrological cycling. Studies have shown that grazing primarily negatively impacts grasslands by reducing species richness and community evenness, whereas enclosures may promote the recovery of species diversity in previously grazed areas, depending on environmental conditions, management history, and the duration of exclusion (Schultz et al., 2011; Hao et al., 2014). Moreover, mowing can affect species growth cycles and competitive strategies by altering grassland productivity and disturbance timing. These contrasting findings highlight the need to move beyond single diversity metrics and to identify mechanistic pathways through which management practices regulate community dynamics and stability.

Methods for assessing community stability have evolved, with common indicators including species diversity indices, population recovery time, and species redundancy (Downing et al., 2020). The relationship between diversity and stability has been widely debated, with some scholars suggesting that high-diversity communities tend to be more stable, as diversity provides species redundancy that helps buffer against external disturbances (Yachi and Loreau, 1999). However, other studies indicate that under certain conditions, high-diversity communities may face intense interspecies resource competition, which could affect community stability (Houlahan et al., 2007). These contrasting perspectives suggest that diversity alone is insufficient to explain stability patterns, and that underlying species dynamics must be explicitly considered.

Species turnover and niche overlap are important mechanisms underlying grassland community stability. A high degree of niche overlap often intensifies interspecies competition, thereby affecting community stability (Prado and Lewinsohn, 2004). Instead, species turnover may either stabilize or destabilize communities depending on the balance between species loss and replacement. Studies have shown that land management practices, such as enclosures, grazing, and mowing, can influence community stability by altering the frequency of species turnover and the degree of niche overlap (Ma et al., 2024).Environmental factors, particularly soil nutrients and climatic conditions, play crucial roles in grassland community stability and species diversity. Soil nutrients, such as organic matter, nitrogen, and phosphorus, influence plant growth and interspecies competition (Wedin and Tilman, 1996). Climatic factors, such as temperature and precipitation, affect species growth cycles, reproductive modes, and competitive relationships both directly and indirectly (Rossignaud et al., 2022). Importantly, plant–soil feedbacks provide a mechanistic link between vegetation dynamics and environmental conditions, as changes in plant composition can modify soil properties, which in turn feed back to influence subsequent community assembly and stability (Liu et al., 2021).

Although numerous studies have revealed the impacts of different land management practices on grassland diversity and stability, research still has certain limitations, particularly in terms of the accumulation of long-term monitoring data, regionally specific studies, and understanding the mechanisms by which different land management practices affect community stability. By explicitly linking species dynamics, plant–soil interactions, and community stability across three contrasting land management practices, this study aims to provide a mechanistic framework for understanding grassland ecosystem responses and to inform sustainable grassland management and conservation strategies.

The Hulunbuir meadow steppe lies in the ecotone between temperate grasslands and forest-steppe, and has highly typical grassland ecological characteristics. It not only possesses high species diversity and a complex community structure but also exerts pivotal ecological roles under the combined pressures of global climate change and land use change (Wang et al., 2022; Legesse et al., 2023). As a representative grassland ecosystem, research on the Hulunbuir meadow steppe is crucial for understanding the response mechanisms, succession patterns, and ecological functions of grassland ecosystems under various disturbances. In this region, grazing and mowing are the primary utilization patterns. While numerous studies have investigated the effects of grazing on grassland community characteristics, there remains a relative lack of comparative research on grassland ecosystems under enclosed, grazing, and mowing management practices.

**Figure 1:**
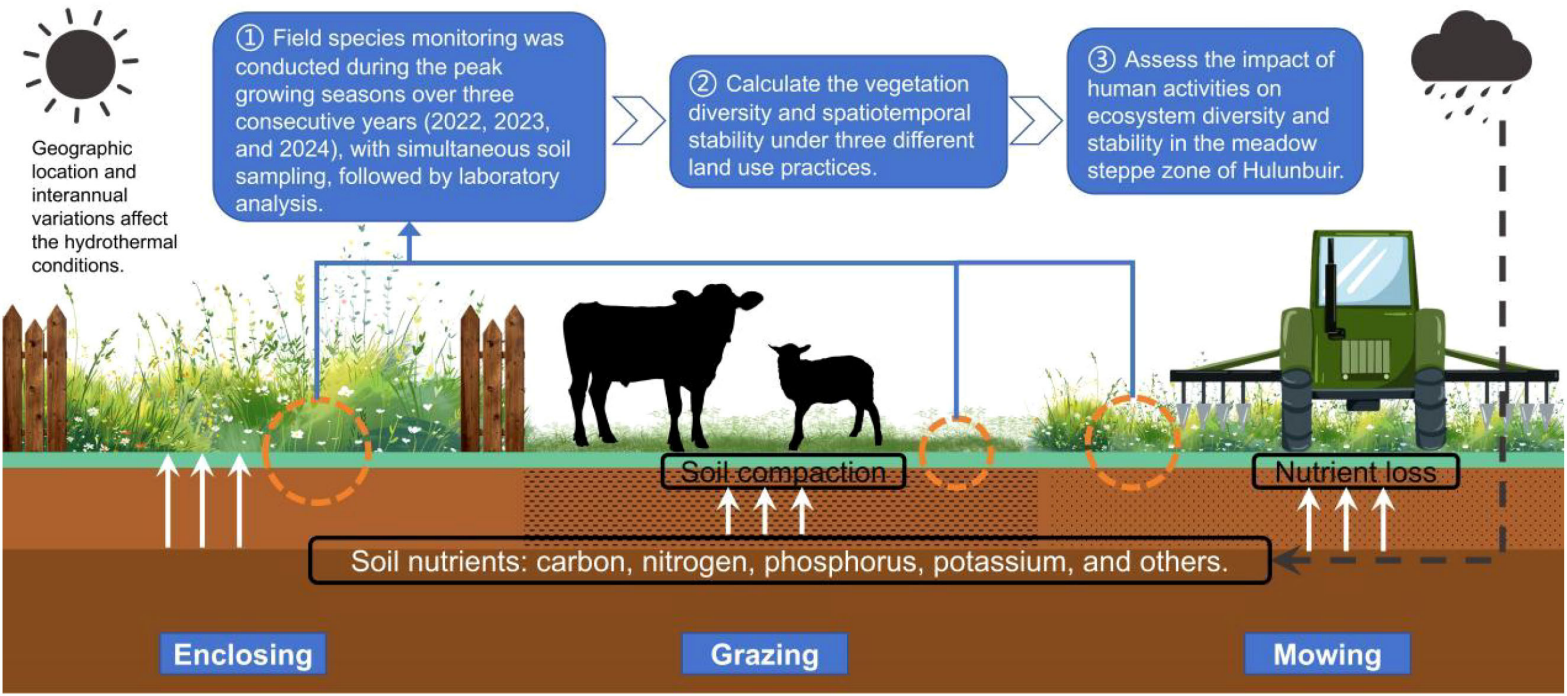
Conceptual map of the research ideas presented in this article.

The main objectives of this study were 1) to examine the effects of different grassland management practices (enclosure, grazing, and mowing) on the dynamic changes in steppe communities, species turnover, and niche overlap in the Hulunbuir grassland and 2) to further analyse the response mechanisms underlying these changes in community diversity and stability. Specifically, we aimed to reveal how different land management practices affect community stability by altering species richness and niche overlap and to explore the role of environmental factors in these processes (**Figure 1**).

## 2 Materials and methods

### 2.1 Overview of the study area

The study area is located in Hulunbuir (115°31′E~126°04′E, 47°05′N~53°20′N), Inner Mongolia. The area is characterized by rolling high plains and low hills, with elevations varying between 600 and 800 m above sea level, and is dominated by chestnut-coloured calcareous soil. The climate is temperate and semihumid, with annual precipitation ranging from 300 to 500 mm, which is concentrated mostly from July through August. The annual average temperatures vary spatially between -2 °C and 4 °C, with January being the coldest month, when temperatures drop below -20 °C, and July being the warmest month, with temperatures ranging between 20 °C and 25 °C. The region’s grassland vegetation is dominated by Poaceae, Asteraceae, and Fabaceae plants, with key dominant species, including *Leymus chinensis*, *Stipa baicalensis*, *Filifolium sibiricum*, *Carex duriuscula*, *Scutellaria baicalensis*, *Allium mongolicum*, *Bupleurum chinense*, and *Cleistogenes squarrosa*.

### 2.2 Experimental design

**Figure 2:**
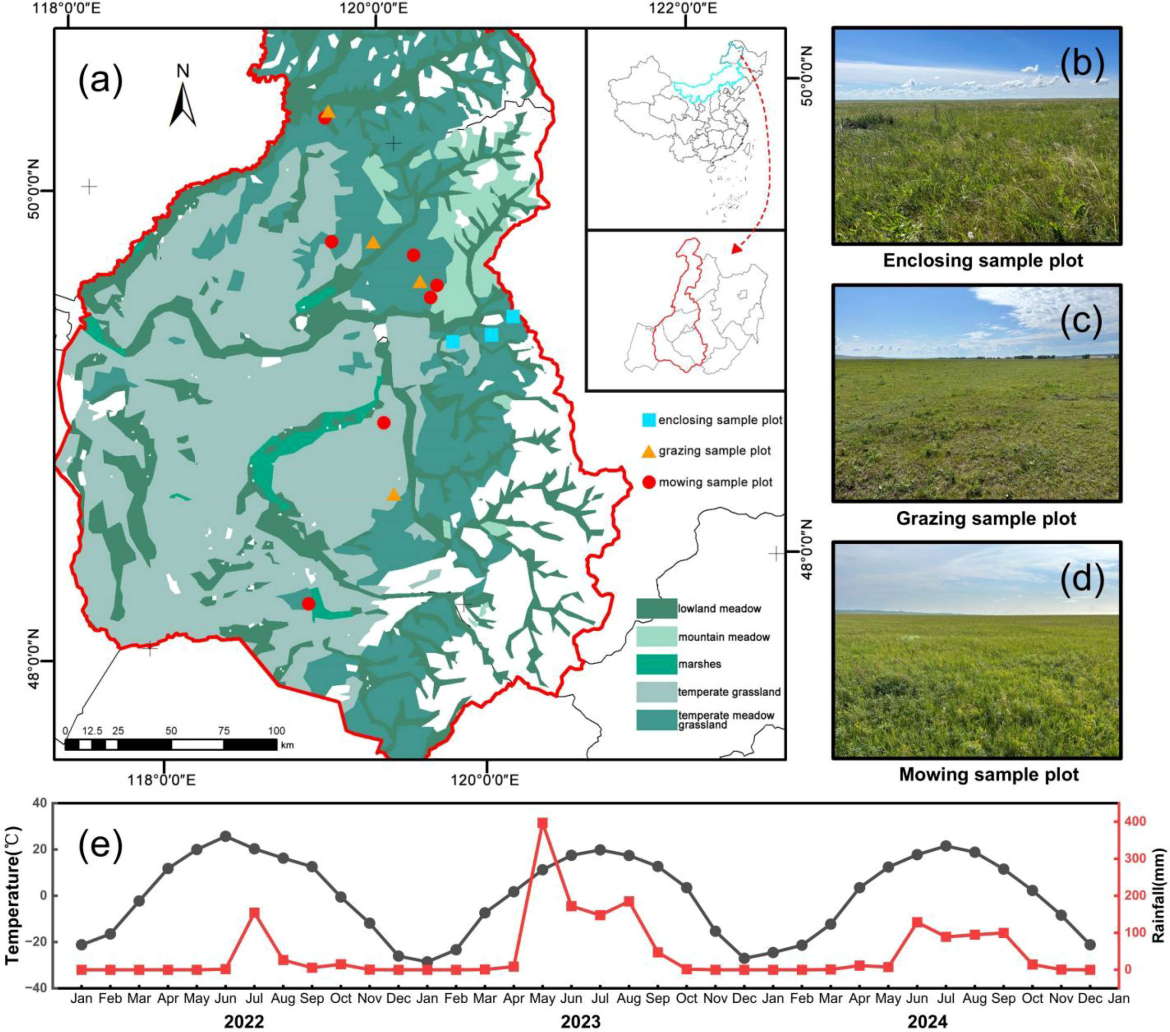
Distribution of plots with enclosure **(b)**, grazing **(c)** and mowing **(d)** practices in temperate meadow grasslands in Hulunbuir **(a)**. Hydrothermal conditions in the monitoring area showed significant seasonal variations over a 3-year period **(e)**.

The experiment was conducted in the temperate meadow steppe of Hulunbuir (**Figure 2**), which focuses on three distinct types of meadow steppe, namely, *L. chinensis* meadow steppe, *S*. *baicalensis* meadow steppe, and F. *sibiricum* meadow steppe, and three land use treatments, namely, enclosure, grazing, and mowing (**Table 1**), were established. Measurements were carried out during the peak growing season (August) from 2022 to 2024. A total of 14 study plots were established, including 3 enclosed plots, 4 grazed plots, and 7 mowed plots (**Figure 2a**). The enclosure plots have been protected from grazing by fencing since 2006, whereas the grazed plots have been managed under locally representative long-term grazing regimes. Mowing was typically conducted once annually during the late growing season after peak biomass accumulation, following local management practices. Disturbance intensity was classified according to the Chinese national standard GB19377 (**Table 1**).

**Table 1:** Specific locations of the experimental sampling points, along with their grassland community types, land management practices, and utilization intensities.

Vegetation type	Plot number	Lon.	Lat.	Elev.	Intensity of use	Land use practice
*Leymus chinensis* meadow steppe	S1	120.180264	49.1140402	620	Nonutilized	enclosure
	S2	120.0398721	49.3851503	628	Moderate	grazing
	S3	120.1356106	49.35691002	662	Light	mowing
	S4	119.525225	49.537393	622.7	Intensive	grazing
	S5	119.511065	49.529603	614.1	Moderate	mowing
*Stipa baicalensis* meadow steppe	S6	120.4202112	49.1261464	657	Nonutilized	enclosure
	S7	120.0989735	49.32852147	635	Light	mowing
	S8	119.66415	48.498648	749.6	Intensive	grazing
	S9	119.668862	48.822133	747.2	Light	mowing
	S10	119.0334083	48.09817615	780	Light	mowing
*Filifolium sibiricum* meadow steppe	S11	120.603576	49.18601788	797	Nonutilized	enclosure
	S12	120.02222	49.5089	756.4	Light	mowing
	S13	119.607617	50.171522	575	Intensive	grazing
	S14	119.607868	50.170922	572	Moderate	mowing

Each study plot covered an area of 0.5 ha. Within each plot, five 1 m × 1 m quadrats were established at the four corners and the centre for the vegetation surveys. Species composition was recorded for each quadrat; natural plant height was measured using a ruler, cover was visually estimated, and density was determined by counting individuals. Aboveground biomass was measured using a species-specific harvesting method: plants were clipped by species and oven-dried at 65 °C to constant weight. Surface litter was collected simultaneously, bagged, transported to the laboratory, dried at 85 °C for 24 h, and weighed. Belowground biomass was sampled using a 30 cm × 30 cm pit-digging method. For clonal species, including dominant grasses, the number of individuals was quantified at the ramet level. In total, 198 quadrats were surveyed over the three years, including 60 in 2022, 68 in 2023, and 70 in 2024.

To further characterize vegetation dynamics from a functional perspective, in addition to taxonomic classification, plant species were grouped into different functional and ecological indicator categories based on life-history traits, forage value, and responses to disturbance. First, species were classified into annual–biennial and perennial plants according to their life-cycle characteristics. High-quality forage species were identified based on grassland utilization standards for the Hulunbuir meadow steppe and relevant regional literature. Degradation indicator species were defined as disturbance-tolerant taxa that tend to increase in abundance under intensive grazing, trampling, or soil compaction and are commonly associated with grassland degradation, following the criteria described in Flora of China (Flora of China Editorial Committee, Chinese Academy of Sciences, 1993). In this study, degradation indicator species mainly included *Potentilla acaulis*, *Cleistogenes squarrosa*, *Euphorbia fischeriana*, and species of *Carex*, which are widely recognized indicators of degraded grassland conditions in northern China. In addition, species were classified into ecological types (xerophytes, mesophytes, and semi-mesophytes) according to their water-use strategies and habitat preferences, following the descriptions in Flora of China. The importance value of each functional or ecological group was calculated as the sum of the importance values of all species belonging to that group.

Following the herbaceous vegetation survey in the quadrats, soil samples were collected along the diagonal of each quadrat via a root auger at three depth intervals: 0–10 cm, 10–20 cm, and 20–30 cm. Three cores were taken from each depth per quadrat, and the samples from each depth were pooled into a single sample, resulting in a total of nine samples per site. Standard methods were applied to determine soil physicochemical properties and nutrient contents, including soil bulk density, soil moisture, pH, organic carbon (OC), total nitrogen (TN), alkali-hydrolyzable nitrogen (AN), total phosphorus (TP), total potassium (TK), available phosphorus (AP), and available potassium (AK).

Meteorological data, including air temperature and humidity, soil temperature, precipitation, wind direction and speed, and evaporation, were recorded at fixed monitoring stations located at each study site.

### 2.3 Index calculation

For diversity, three dimensions—α, β, and γ—are calculated. The α diversity of the community is represented by the following indices: the Shannon–Wiener diversity index (H’), Simpson diversity index (D), Pielou evenness index (J’), and Margalef richness index (d):

(1) $$\text{H'}=-\sum_{i=1}^{\text{S}}{\text{p}}_{i}\ln\left({\text{p}}_{i}\right)$$

(2) $$\begin{array}{c} \text{D}=\sum_{i=1}^{\text{S}}{\text{p}}_{i}^{2} \end{array}$$

(3) $$\text{J'}=\frac{\text{H'}}{\ln\left(\text{S}\right)}$$

(4) $$\begin{array}{c} \text{d}=\frac{\text{S}-1}{\ln(\text{N})} \end{array}$$

where:

H′= Shannon–Wiener diversity index.

D= Simpson diversity index.

J′= Pielou evenness index.

d= Margalef richness index.

S= total number of species.

N= total number of individuals across all species.

p*_i_*= relative abundance of the i-th species, defined as the proportion of the i-th species’ individuals to the total number of individuals.

The Whittaker diversity index (β_w_) was used to measure the community’s β diversity. The Jaccard index (J) was calculated to indicate the overlap in ecological niches between species in different plots and was used to assess the patterns of species interactions under different land use treatments. Additionally, the alternating species composition under different years and land use treatments was quantified as follows:

(5) $$\begin{array}{c} {\text{β}}_{\text{w}}=\frac{S_{T}}{\bar{S}} \end{array}$$

(6) $$\begin{array}{c} \text{J}=\frac{\left|\text{A}\cap \text{B}\right|}{\left|\text{A}\cup \text{B}\right|} \end{array}$$

where:

βw= Whittaker diversity index.

S_T_= total number of species across all plots in the study area (i.e., species richness for the entire region).

$\bar{S}=$ average number of species per plot (i.e., the mean species richness across all plots).

J= Jaccard index.

A and B represent two plots or species sets.

∣A∩B∣= size of the intersection between sets A and B, i.e., the number of species common to both sets.

∣A∪B∣= size of the union between sets A and B, i.e., the total number of species in both sets.

γ diversity:

(7) $$\begin{array}{c} \gamma =\alpha \times \beta \end{array}$$

In this study, α diversity was represented by the Margalef richness index, and β diversity was represented by the Whittaker diversity index.

The community stability index is represented by the coefficient of variation (CV) of community biomass. The coefficient of variation is a standardized statistic used to measure the relative fluctuation or dispersion of community biomass:

(8) $$\begin{array}{c} \text{CV}=\frac{\sigma }{\text{μ}} \end{array}$$

where:

σ = standard deviation of community biomass.

μ= mean community biomass.

### 2.4 Data processing

Data processing and graphical representations were performed via Excel 2023 and Origin 2024 software. All the statistical analyses were conducted via SPSS 20.0 for Windows (USA) and R software (version 4.3.2, R Development Core Team), with the statistical significance set at α=0.05. To minimize subjective sampling errors, outliers in the vegetation quadrat data (defined as values exceeding twice the standard deviation from the mean) were excluded prior to calculating the diversity and stability indices. In total, 30 observations (2.75% of the total dataset) were removed following this criterion. In R, the vegan package was used to calculate diversity indices, and ggplot2 was used for data visualization.

One-way analysis of variance (ANOVA) was used to examine differences in community structure under different land management practices. Where applicable, Duncan’s multiple range test was applied to illustrate groupings among treatments. Statistical significance was determined based on the omnibus ANOVA at p < 0.05. To assess the effects of interannual variation, treatment, and their interaction on plant community characteristics, two-way ANOVA was performed. In this model, interannual variation was considered a main effect, and the treatment (i.e., land management practice) was also treated as a main effect. Additionally, the interannual × treatment interaction was included to investigate whether the effects of land management practices varied across different years. The aim of this approach was to evaluate both the independent and combined effects of these factors on the observed community traits. To estimate the overall overlap in ecological niches among plots under different land management practices, the Jaccard index was calculated for each plot and averaged across all plots within each treatment. A nonlinear regression model was used to explore the relationship between species turnover and community stability.

Pearson and Spearman correlation analyses were applied to investigate the relationships among diversity, stability, vegetation factors, and environmental factors. Pearson correlation was used for normally distributed data, whereas Spearman correlation was applied for nonnormally distributed data. These analyses provided insights into the interrelationships among various ecological factors. This method helps elucidate how community traits, such as species richness and community structure, are related to environmental variables (e.g., soil nutrients and climate) and provides further understanding of how environmental factors shape plant community dynamics.

Structural equation modelling (SEM) was used to assess the direct and indirect effects of environmental factors on plant diversity and community stability under different land management practices. SEM analyses were performed using AMOS 21.0 (AMOS Development Co.), following an *a priori* conceptual model. Path coefficients were estimated via maximum likelihood estimation, and model fit was assessed via χ^2^, degrees of freedom (df), the χ^2^/df ratio, RMR, and p values.

## 3 Results

### 3.1 Structural characteristics of grassland plant communities under different land management practices

**Figure 3:**
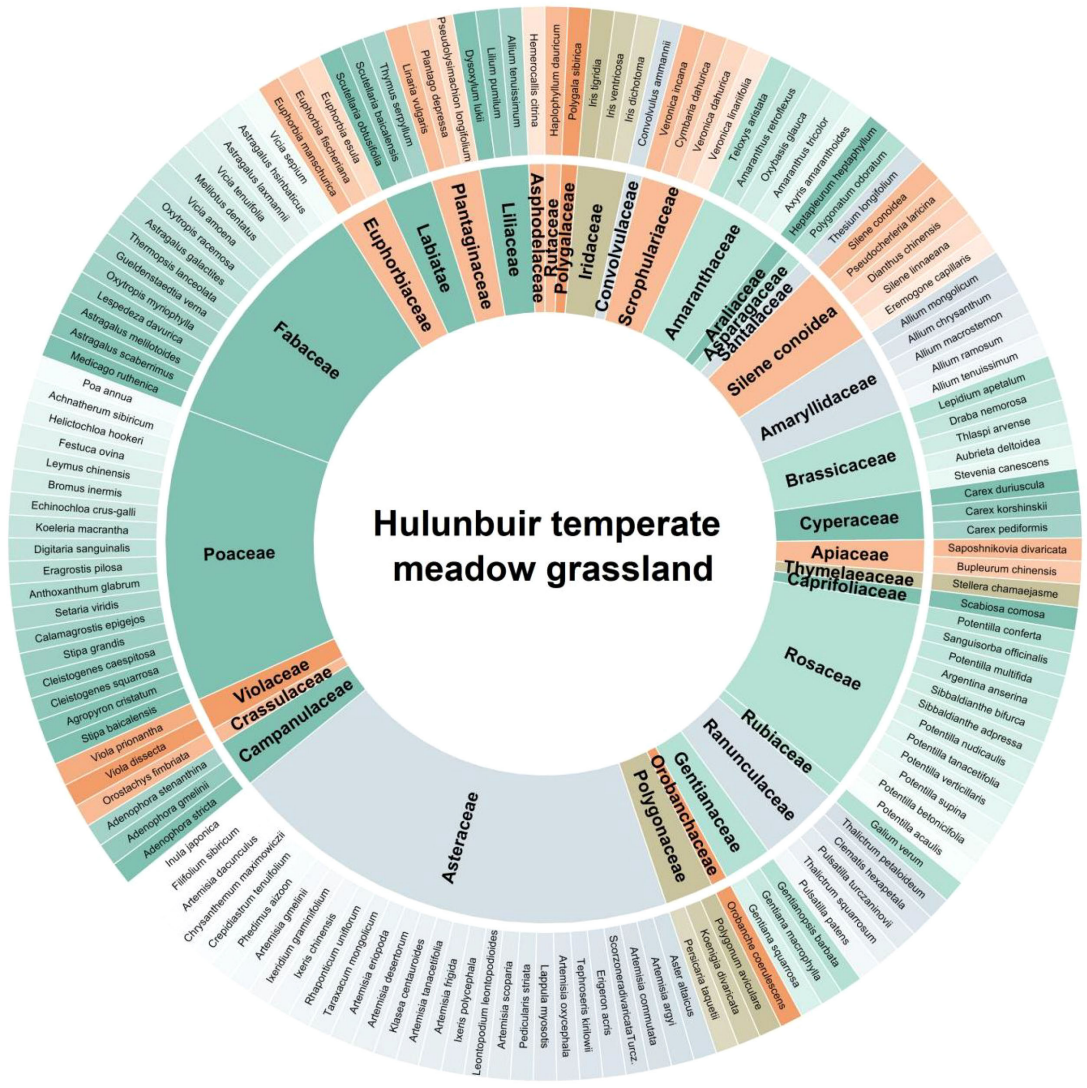
All the species monitored in the temperate meadow grassland sampling sites in Hulunbuir during August 2022, 2023, and 2024 and their classification into family-level plant groups.

During the three-year observation period from 2022 to 2024, a total of 145 plant species belonging to 33 families—primarily *Poaceae, Asteraceae, Fabaceae, Cyperaceae*, and *Rosaceae*—were recorded across meadow steppe sites in the Hulunbuir region (**Figure 3**).

**Figure 4:**
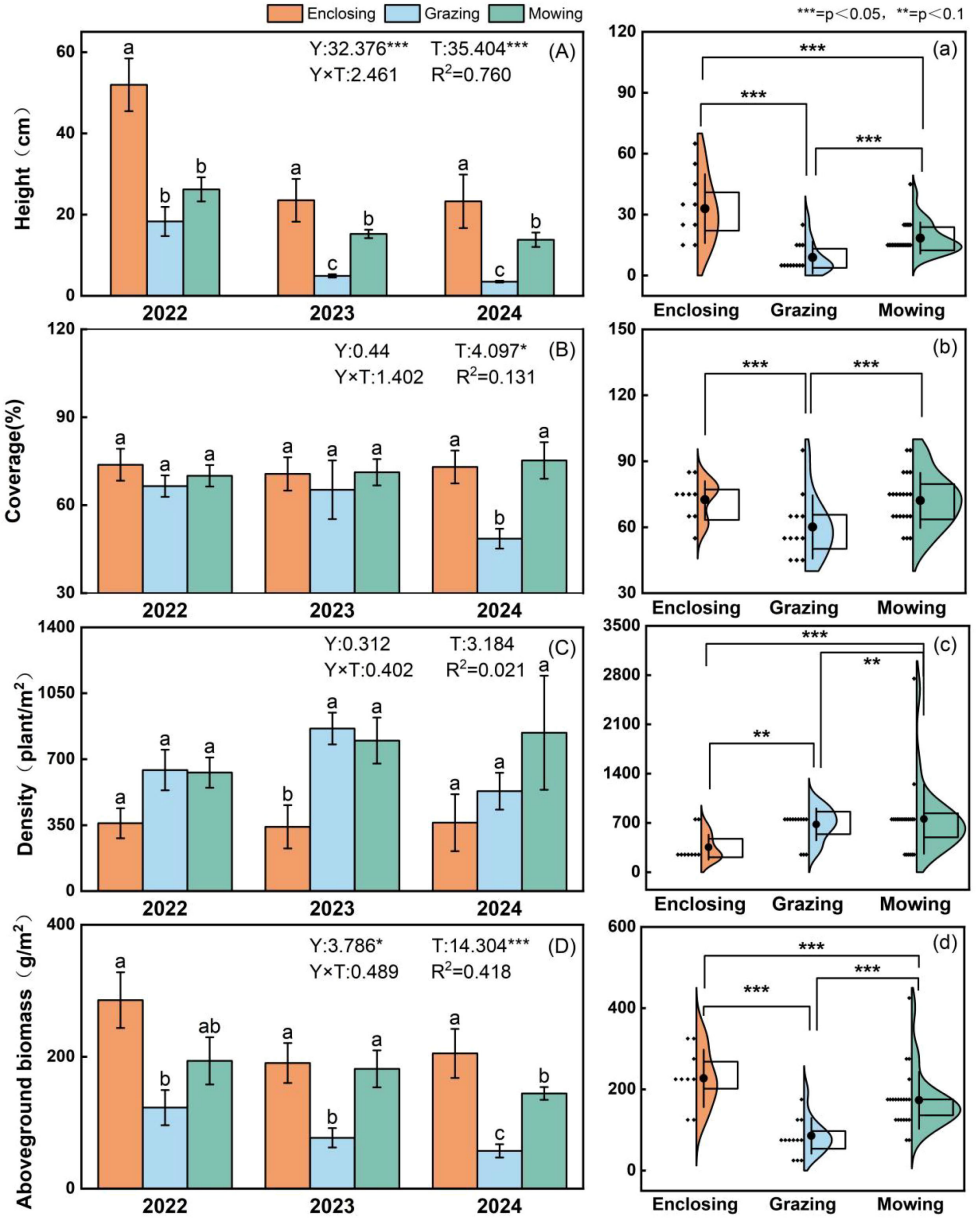
Effects of three different land management practices in 2022, 2023, and 2024 on the height **(A)**, cover **(B)**, density **(C)**, and biomass **(D)** characteristics of the aboveground plant community were analysed via variance analysis for each year and each treatment group. The violin plots represent the distributions and differences in the aboveground plant community height **(a)**, cover **(b)**, density **(c)**, and biomass **(d)** based on the combined data from the three years of monitoring. The bar charts show the mean values (± SE) of each indicator under the three land management practices for each year, with different lowercase letters indicate groupings based on Duncan’s multiple range test. Y: interannual main effects; T, treatment main effects; Y×T, interannual×treatment interaction effects. * indicates significance at the 0.05 level (p<0.05); ** indicates significance at the 0.01 level (p<0.01); *** indicates significance at the 0.001 level (p<0.001).

The structural characteristics of plant communities—including plant height, cover, density, and aboveground biomass—were significantly influenced by land-use practices (enclosure, grazing, and mowing) throughout the study period. Over the three years of monitoring, plant density was significantly higher under mowing than under grazing and enclosure (**Figure 4c**; **Supplementary Table S6**), whereas enclosure significantly increased plant height and aboveground biomass (**Figures 4A, D**; **Supplementary Tables S1**, **S2**, **S7**, **S8**; p < 0.001). Interannual variation also had a significant effect on plant height (p < 0.001), indicating that this trait may be highly sensitive to year-to-year fluctuations in precipitation and temperature.

Although no significant differences in plant density were observed among land-use treatments overall, a marginal land-use effect on plant density was observed in the enclosed plots in 2023 (**Figure 4C**; **Supplementary Table S5**; F = 3.861, p = 0.054). Consistent with this trend, plant density under enclosure tended to be lower than under grazing and mowing in 2023. This year-specific response is likely associated with the substantially higher precipitation in 2023 compared with 2022 and 2024. In long-term enclosed systems, shading effects and competitive exclusion by tall perennial species often constrain the establishment of understory vegetation, whereas livestock grazing and mowing activities can increase vegetation fragmentation, thereby promoting the formation of more plant tussocks and ramets (Wang et al., 2023; Hassan and Wang, 2024).

**Figure 5:**
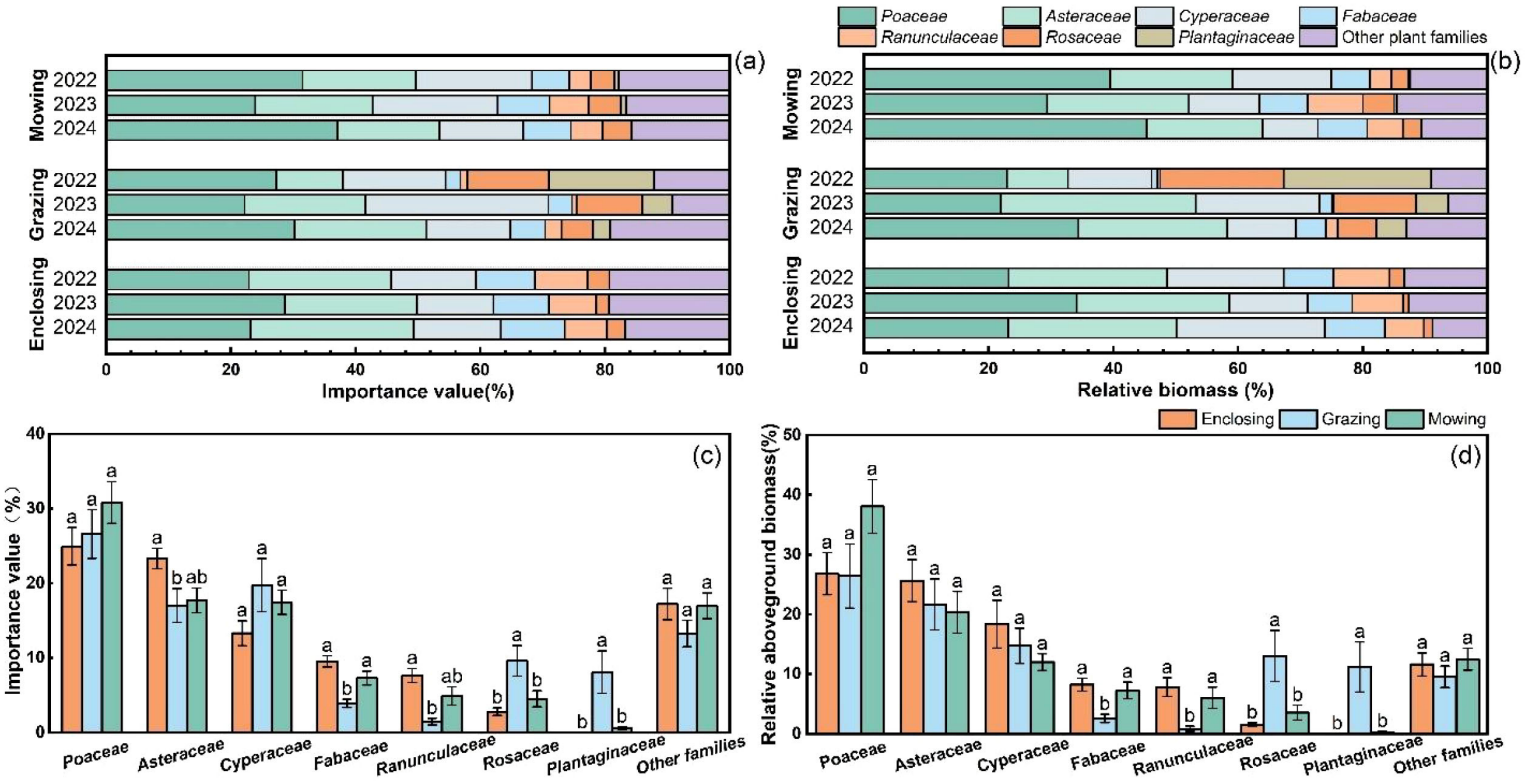
Importance value **(a)** and relative biomass **(a)** of each family, calculated based on the plant functional group classification under different land management practices from 2022 to 2024. The stacked bar chart represents the proportion of each plant family in the grassland community, whereas the bar charts display the mean importance value (± SE) **(c)** and mean relative biomass (± SE) **(d)** of each family over the three years. Different lowercase letters indicate groupings based on Duncan’s multiple range test. Plant families shown represent taxa with similar functional traits and disturbance-response strategies, and are used to illustrate functional shifts in community composition.

Plant functional group composition differed markedly among the three land-use regimes (enclosure, grazing, and mowing) (**Figure 5**; **Supplementary Tables S9**, **S10**). Plant families are presented here as representative taxa characterized by similar functional traits and disturbance-response strategies. Overall patterns of importance values were consistent with aboveground biomass, with Poaceae dominating across all land-use types, followed by Asteraceae and Cyperaceae.

Grazing significantly reduced the importance values and relative biomass of grazing-sensitive forb taxa (e.g., Asteraceae and Fabaceae), while increasing those of disturbance-tolerant taxa such as Rosaceae and Plantaginaceae. Among these, Rosaceae showed high sensitivity to land-use change, whereas Plantaginaceae exhibited a significant year × treatment interaction, indicating joint regulation by interannual variability and grazing disturbance (**Table 2**). These results support the conclusion that grazing favors trampling-tolerant indicator taxa. Under enclosure, grazing-sensitive forb taxa exhibited higher importance values and relative biomass than under grazing and mowing, whereas under mowing, Poaceae showed increased dominance, likely due to their strong tillering capacity and compensatory growth following cutting.

**Table 2:** Interactive analysis of the importance values of plant functional groups under different land management practices in the temperate meadow grassland of Hulunbuir.

Function	Factor	F	Significance
Poaceae	Interannual	0.662	0.523
	Treatment	1.029	0.369
	Interannual×Treatment	0.75	0.565
Asteraceae	Interannual	1.037	0.366
	Treatment	2.494	0.098
	Interannual×Treatment	1.271	0.301
Cyperaceae	Interannual	2.25	0.121
	Treatment	1.601	0.217
	Interannual×Treatment	1.285	0.296
Fabaceae	Interannual	0.927	0.406
	Treatment	7.179	0.003**
	Interannual×Treatment	0.314	0.866
Ranunculaceae	Interannual	0.057	0.945
	Treatment	5.017	0.013*
	Interannual×Treatment	0.38	0.821
Rosaceae	Interannual	0.785	0.464
	Treatment	5.167	0.011*
	Interannual×Treatment	1.019	0.412
Plantaginaceae	Interannual	4.387	0.020*
	Treatment	13.47	<0.001***
	Interannual×Treatment	4.209	0.007**
Other plant families	Interannual	0.284	0.754
	Treatment	1.27	0.294
	Interannual×Treatment	1.008	0.417

* indicates significance at the 0.05 level (p<0.05); ** indicates significance at the 0.01 level (p<0.01); *** indicates significance at the 0.001 level (p<0.001).

**Figure 6:**
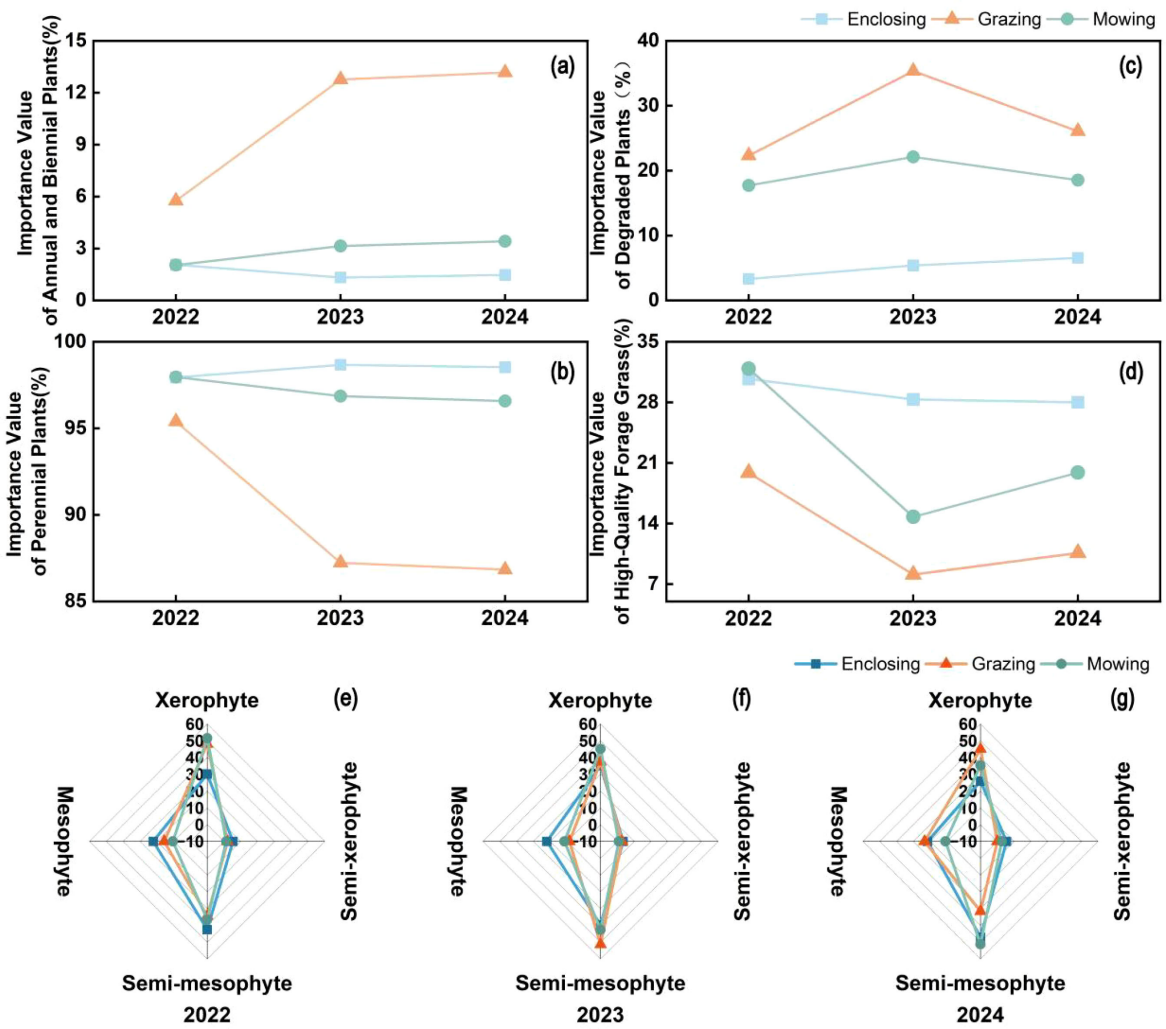
Comparison of the life form and ecological type trends from 2022 to 2024, including a comparison of the variation in importance values of annual and biennial plants **(a)**, perennial plants **(b)**, indicator species for degradation **(c)**, and high-quality forage species **(d)**, as well as the proportion of species by ecological type **(e–g)** across each year.

The results showed that the interannual variation patterns of annual and biennial plants were consistent with those of the importance values of degradation indicator species (**Figures 6a, c**). The proportions of both groups were significantly higher in grazed plots than in mown and enclosed plots. Notably, the proportions of annual–biennial plants and degradation indicator species in the grazed plots exhibited a continuous increasing trend over the three-year period. In contrast, the dynamics of perennial species were consistent with those of the importance values of high-quality forage groups (**Figures 6b, d**). The relative abundances of both groups were consistently highest in the enclosed plots, while the proportions of these two groups declined across all three land-use types over the three-year period.

The ecological type composition of plant communities in the enclosed plots remained relatively stable, being mainly composed of xeromesophytic and mesophytic species, with the proportion of mesophytes remaining largely unchanged throughout the study period. Grazing increased the proportion of xerophytic species, whereas the mown plots were dominated by xeromesophytes and xerophytes, showing an intermediate composition between the enclosed and grazed treatments (**Figures 6e–g**). These results further indicate that intensive grazing accelerates the xerophytization process of grassland plant communities and promotes ecological degradation.

### 3.2 Characteristics of grassland plant diversity and community stability under different land use practices

**Figure 7:**
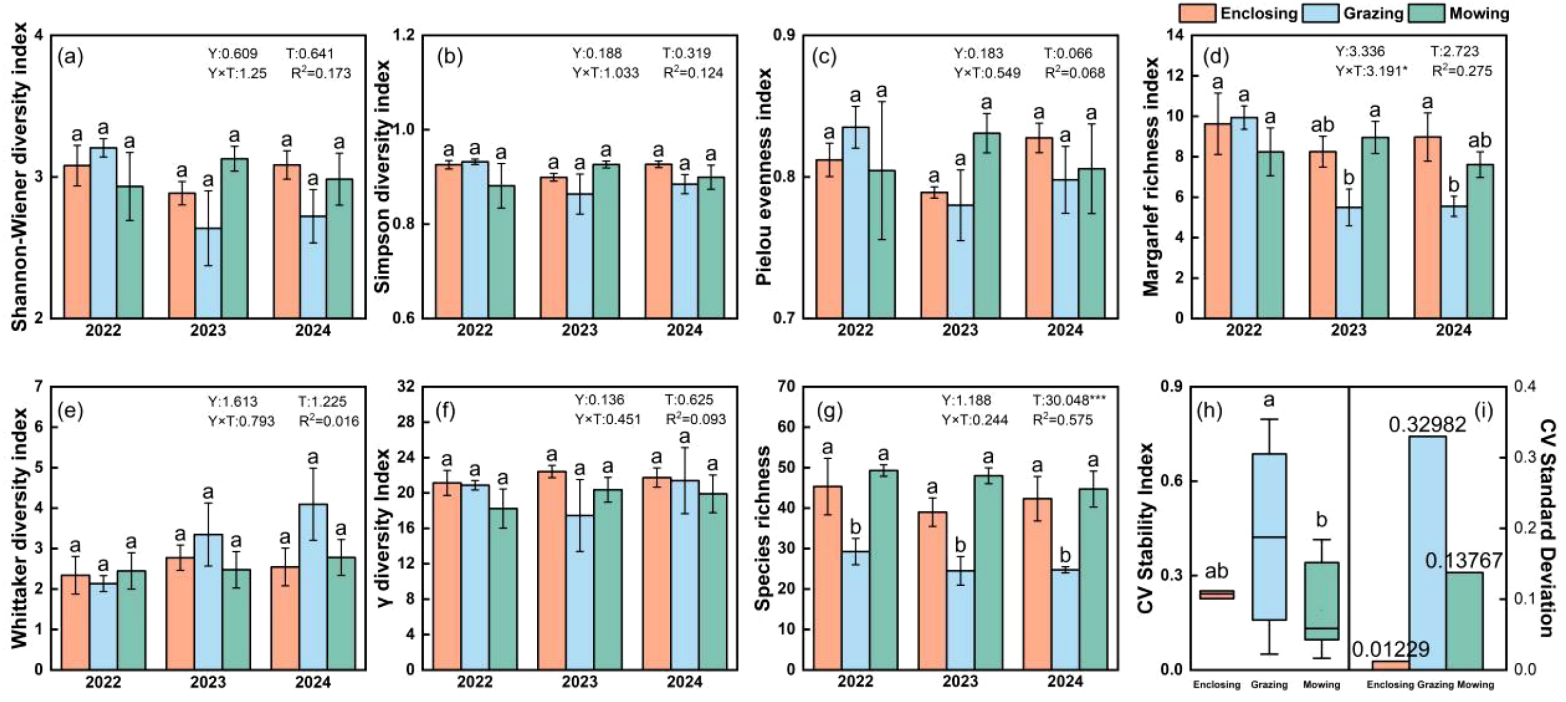
Effects of enclosure, grazing, and mowing land management practices from 2022 to 2024 on plant diversity, including α diversity **(a–d)**, β diversity **(e)**, and γ diversity **(f)**. The bar charts represent the mean diversity values (± SE) for each land use practice. The box plots **(h)** illustrate the coefficient of variation for each land use practice over the three years, reflecting the spatiotemporal stability of the monitored areas. The final bar chart **(i)** shows the standard deviation of the coefficient of variation for each land use practice, indicating the degree of data dispersion. Different lowercase letters indicate groupings based on Duncan’s multiple range test. Y, Interannual main effects; T, treatment main effects; Y×T, interannual×treatment interaction effects. * indicates significance at the 0.05 level (p<0.05); ** indicates significance at the 0.01 level (p<0.01); *** indicates significance at the 0.001 level (p<0.001).

Among the diversity metrics examined, the Margalef richness index showed a significant response to land-use treatments, with values under grazing being significantly lower than those under enclosure and mowing (p < 0.05) (**Figures 7d, g**; **Supplementary Tables S14**, **S17**). In addition, a significant interaction effect between year and treatment was detected for the Margalef index (p < 0.05), whereas treatment exerted a highly significant main effect on species richness (p < 0.001). These results indicate that grazing reduces plant species richness and diversity in grassland ecosystems. Notably, species richness in mown plots exceeded that in enclosed plots, which may be attributed to the reduction in the dominance of competitive species by mowing, thereby creating opportunities for subordinate species to persist and coexist. In contrast, no significant differences were detected among land-use types for the Shannon–Wiener index, Simpson index, Pielou evenness index, or γ diversity (**Figures 7a–c, f**; **Supplementary Tables S11**-**S13**, **S15**, **S16**). The lack of significant treatment effects likely reflects substantial spatial heterogeneity among plots and pronounced interannual climatic variability during the study period.

The contrasting responses between the Margalef index and the other diversity metrics reflect fundamental differences in the ecological attributes captured by these indices. Specifically, the Margalef index primarily quantifies species richness and is more sensitive to changes in rare or subordinate species, whereas the Shannon–Wiener, Simpson, Pielou evenness, and γ-diversity indices integrate species relative abundance and community structure, and thus more strongly reflect dominance patterns and community stability (Lamb et al., 2009; Bollarapu et al., 2024). Consequently, grazing-induced reductions in species richness can be detected by the Margalef index without producing parallel changes in abundance-weighted diversity metrics.

Community stability was assessed using the coefficient of variation (CV) of aboveground biomass over the three-year period. The results showed that both the mean CV and its standard deviation were significantly greater under grazing than under enclosure and mowing (p < 0.05) (**Figure 7h**), indicating reduced temporal stability in grazed communities. A bar chart (**Figure 7i**) presented the standard deviation of CV values for each treatment and uses it as a proxy for spatial stability at the landscape scale. The results demonstrated that grazing led to lower community stability in both temporal and spatial dimensions. Although mown plots exhibited greater temporal stability than enclosed plots, the large dispersion of values among sites suggests relatively weaker spatial stability. Overall, the integrated assessment of community stability ranked the land-use types as follows: enclosure > mowing > grazing.

### 3.3 Impact of species turnover and niche overlap on stability

**Figure 8:**
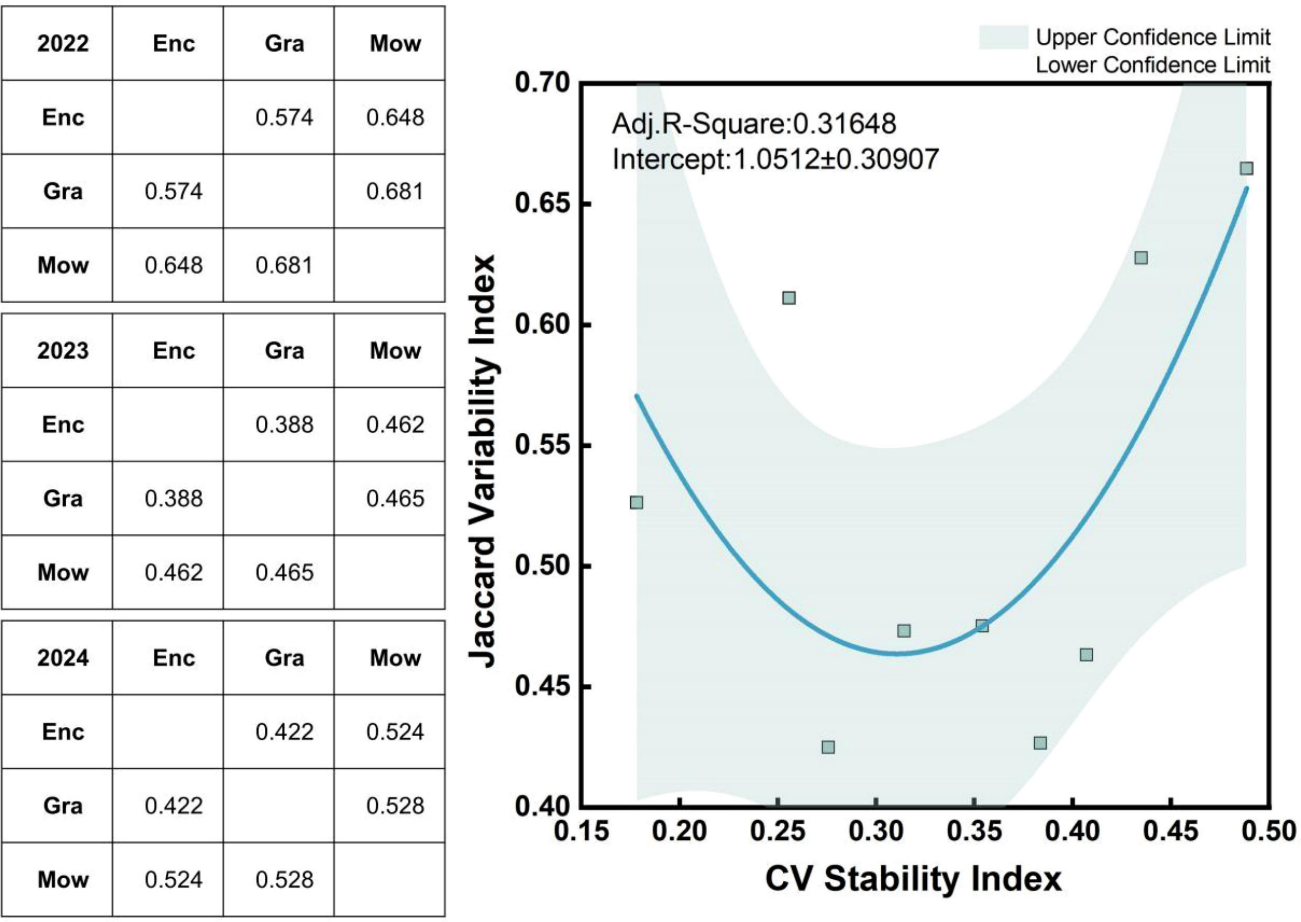
The table on the left presents the Jaccard similarity index calculated for each year, with a comparison of two land management practices to represent the niche overlap between sampling sites. There is a noticeable nonlinear relationship between niche overlap and community stability. The green points represent data groups corresponding to different land management practices for each year.

The table on the left (**Figure 8**) presents the annual Jaccard similarity indices, which were calculated to evaluate the degree of ecological niche overlap between sampling sites under different grassland use regimes. The results revealed clear differences in niche overlap among the three treatments. In 2022, the similarity index between enclosure and mowing was 0.648, whereas the index between grazing and enclosure was lower, at 0.574. This finding indicates that grazed sites shared fewer species with enclosed sites, reflecting reduced ecological similarity, whereas mown sites presented a greater degree of overlap with enclosed communities. Mowing thus represented an intermediate state between enclosure and grazing in terms of community composition. Although the similarity indices fluctuated slightly over the study period, they generally remained within a range of 0.38~0.68 across years, suggesting moderate but variable ecological overlap driven by land use and interannual environmental variation.

The overall niche overlap values for each land use type were calculated based on the Jaccard index. As the CV stability index increased, the Jaccard variation index exhibited a nonlinear relationship. Specifically, when the Jaccard index was low (approximately 0.45, indicating low niche overlap), the CV stability index ranged from 0.25 to 0.35, reflecting lower community stability. As the Jaccard index increased, the CV stability index either increased or decreased, with a Jaccard index of 0.45 serving as a critical threshold (**Figure 8**).

### 3.4 Relationships between plant community characteristics and environmental factors

**Figure 9:**
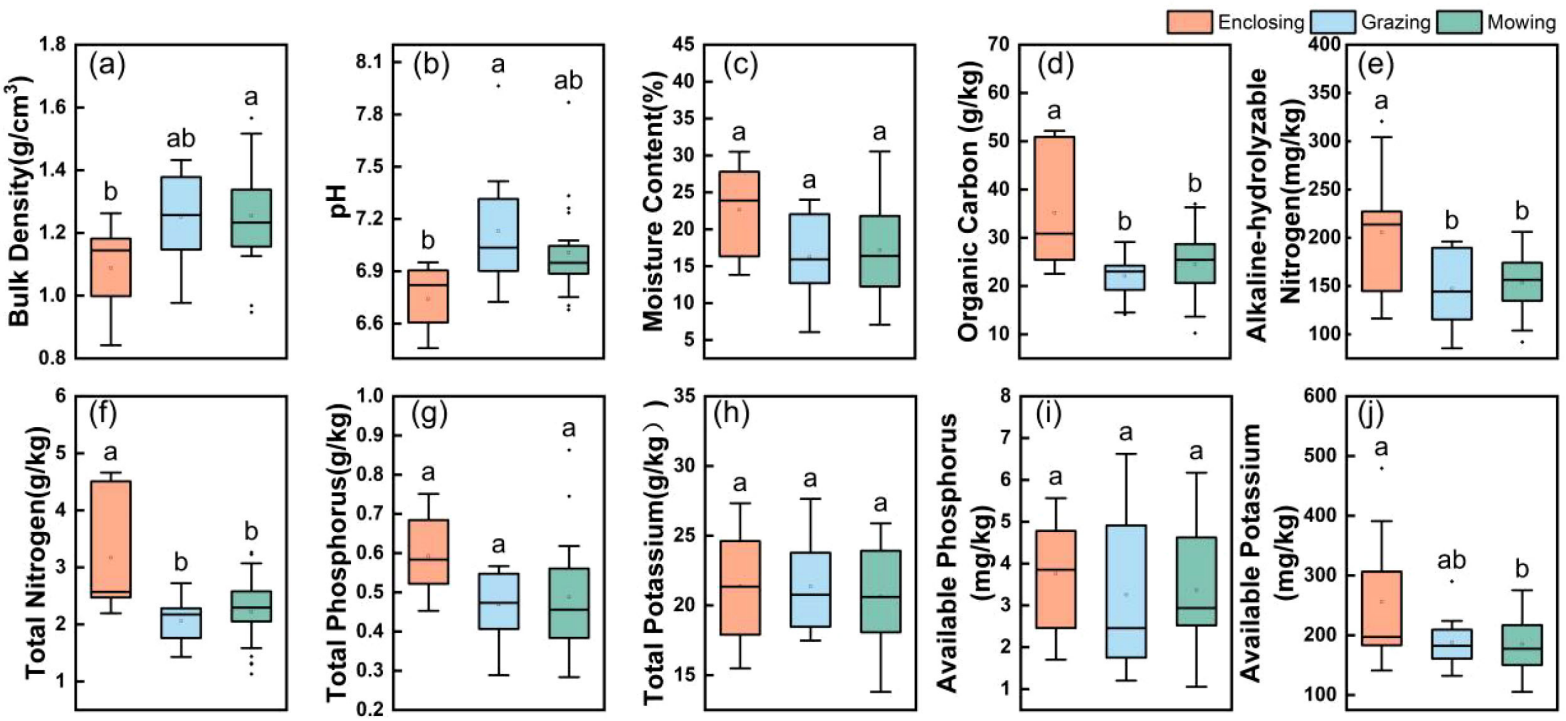
Soil physicochemical properties **(a–c)** and nutrient content distributions **(d–j)** under the three land management practices. The figures display the mean values (± SE) of the measured data from 2022 to 2024, with different lowercase letters indicate groupings based on Duncan’s multiple range test.

As shown in **Figure 9**, significant differences in soil physicochemical properties and nutrient levels were observed among the three land use regimes. The soil bulk density and pH varied markedly across the treatments. Specifically, the bulk density in mown plots was slightly greater than that in grazed plots, whereas enclosed plots presented substantially lower bulk density than both. The soil acidity followed a pattern of enclosure > mowing > grazing (p < 0.05), indicating pronounced soil compaction and acidification under grazing conditions.

Soil organic carbon, alkali-hydrolyzable nitrogen, total nitrogen, and available potassium contents in enclosed plots were significantly higher than those in grazed and mown plots (**Figures 9d-f, j**; **Supplementary Table S18**, P < 0.05). These results indicated that grazing and mowing were associated with greater losses of key soil nutrients. In contrast, there were no significant differences for soil moisture, total phosphorus, total potassium, and available phosphorus among treatments(**Figures 9c, g-i**; **Supplementary Table S18**). The absence of significant differences in these variables may reflect strong spatial heterogeneity among plots, temporal variability in precipitation, and the relatively slow response of some soil properties to land management at the temporal scale of this study.

**Figure 10:**
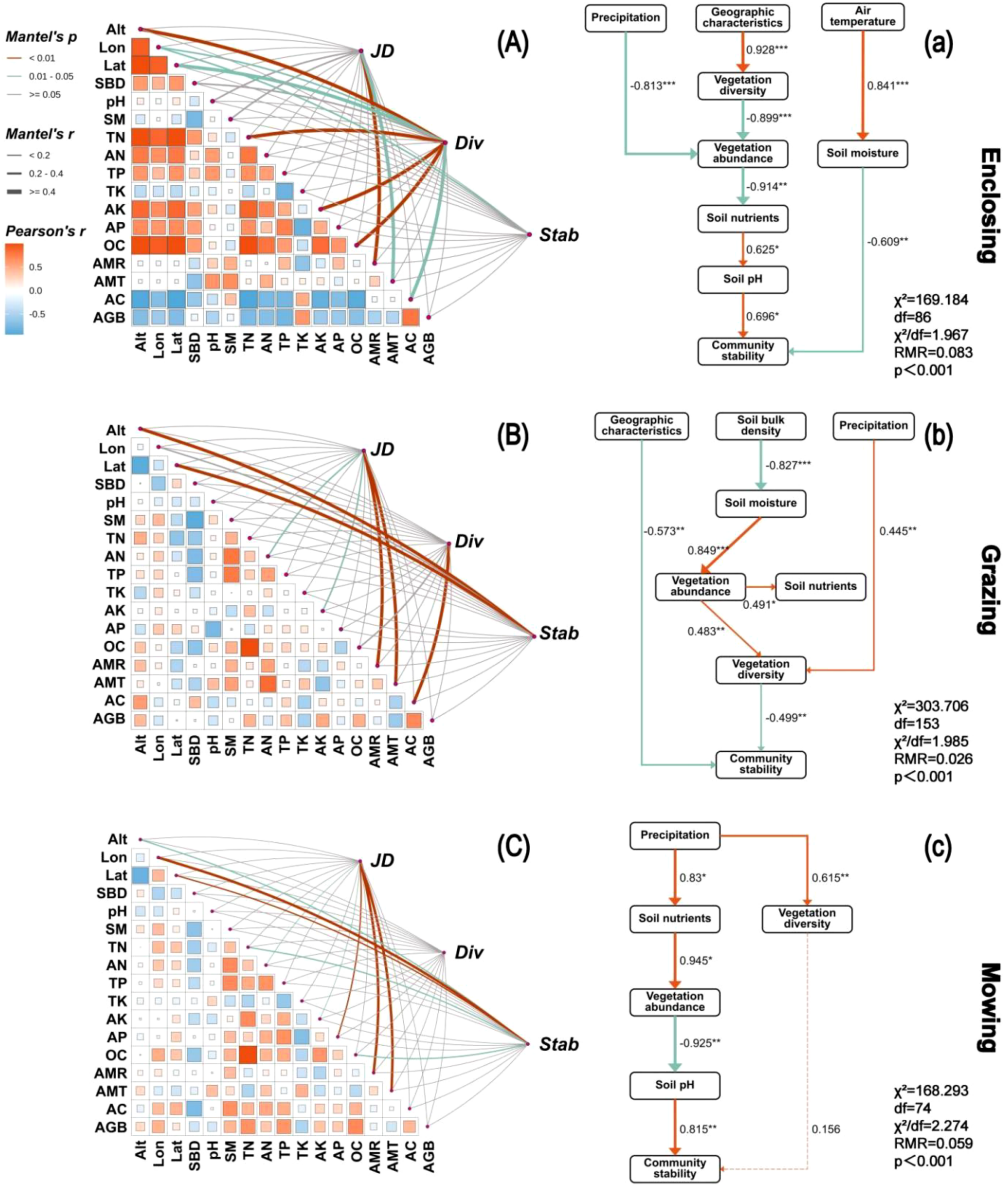
Mantel test analysis of plant diversity, community stability, and various ecosystem factors under enclosure **(A)**, grazing **(B)**, and mowing **(C)** conditions in the temperate meadow grassland of Hulunbuir. The colour gradient represents Spearman’s correlation coefficients of pairwise comparisons between ecosystem functions. The edge width represents correlations, and the edge colour denotes the significance. (JD, Jaccard index; Div, diversity index; Stab, stability index; Alt, altitude; Lon, longitude; Lat, latitude; SBD, soil bulk density; pH, soil pH; SM, soil moisture; TN, total nitrogen; AN, alkaline hydrolysable nitrogen; TP, total phosphorus; TK, total potassium; AK, available potassium; AP, available phosphorus; OC, organic carbon; AMR, monthly average precipitation; AMT, monthly average temperature; AC, community coverage; AGB, aboveground biomass.) Final piecewise structural equation modelling results based on the three-year observational monitoring dataset under enclosure **(a)**, grazing **(b)**, and mowing **(c)** conditions. Pathways of how plant diversity and the presence of functional groups in ecosystems influence spatial and temporal community stability are shown. The orange arrows represent significant positive pathways. The line thickness is proportional to the strength of the relationship. The numbers on the arrows are standardized path coefficients (scaled by their means and standard deviations), and the asterisks indicate statistical significance (***p < 0.001; **p < 0.01; *p < 0.05).

Structural equation modelling (SEM) revealed distinct pathways influencing plant diversity and community stability under different land use regimes (**Figures 10A–C**). Under the enclosure regime (**Figure 10A**), plant diversity was significantly associated with geographic location, soil nutrients, and community abundance (p < 0.01), whereas community stability was mainly influenced by geographic location (p < 0.05). Geographic location had a strong positive effect on species richness (path coefficient = 0.928, p < 0.001). Precipitation was negatively correlated with vegetation abundance (path coefficient = –0.813, p < 0.001) and indirectly affected community stability through its influence on soil pH (path coefficient = 0.696, p < 0.05). Meanwhile, growing-season temperature increased soil moisture (path coefficient = 0.841, p < 0.001), which in turn exerted a negative effect on community stability (path coefficient = –0.609, p < 0.01).

Under the grazing regime (**Figure 10B**), plant diversity was significantly correlated with vegetation abundance (p < 0.01), and community stability was significantly associated with elevation and geographic location (p < 0.01). Soil bulk density had significant positive effects on both soil moisture and vegetation abundance and, together with precipitation, promoted an increase in plant diversity (path coefficients = 0.483 and 0.445, respectively; p < 0.01). However, both geographic location and plant diversity exerted significant negative effects on community stability (path coefficients = –0.573 and –0.499, respectively; p < 0.01).

Under the mowing regime (**Figure 10C**), no significant pathway relationships were detected between plant diversity and environmental variables, whereas community stability remained significantly associated with elevation and geographic location (p < 0.01). Precipitation exerted a significant positive effect on community stability through its influence on soil pH (path coefficient = 0.815, p < 0.01).

## 4 Discussion

### 4.1 Differences in grassland plant communities under different land use practices

Across the temperate meadow steppes of Hulunbuir, our results demonstrated that enclosure, grazing, and mowing exerted contrasting effects on grassland plant communities. Enclosure generally promoted vegetation recovery, leading to greater plant height, aboveground biomass, and higher proportions of perennial and high-quality forage species. In contrast, grazing reduced community stature and stability and increased the dominance of annual plants and degradation indicator species, whereas mowing produced intermediate responses between these two extremes. Together, these patterns indicated that grassland communities respond strongly to the intensity and type of disturbance, with clear consequences for both structure and stability.

These findings are broadly consistent with previous studies showing that enclosure enhances vegetation recovery by reducing anthropogenic disturbance and promoting natural succession (Seymour et al., 2010; Liu et al., 2023). Under relatively favorable water and nutrient conditions, such positive effects are further amplified through improvements in soil structure and microbial activity (Guo et al., 2019; Wu et al., 2022; Zhao et al., 2024). Similarly, the negative impacts of grazing observed in our study align well with earlier reports that herbivory reshapes plant growth patterns and species composition (Price et al., 2022), while overgrazing favors drought-, cold-, and trampling-tolerant species at the expense of community diversity and ecosystem functioning (Shen et al., 2021). In addition, selective feeding by livestock reduces the abundance of leguminous species (Su et al., 2023), whereas trampling increases the prevalence of *Plantago* species (Ludvikova et al., 2014). Beyond direct biomass removal, grazing-induced soil compaction—reflected by increased soil bulk density—further constrains root growth, water infiltration, microbial activity, and nutrient cycling, thereby weakening stabilizing plant–soil feedbacks and accelerating community degradation. In contrast, mowing reduces herbivory pressure while maintaining relatively diverse species assemblages (Collins et al., 1998), and the regular removal of senescent biomass can promote the establishment of new individuals and help sustain community diversity and structural stability (Zhang et al., 2023).

However, not all previous studies report consistently positive effects of enclosure on diversity and stability. Several investigations have shown that long-term enclosure may lead to declines in both diversity and stability due to intensified interspecific competition and altered nutrient dynamics (Lu et al., 2021; Daleo et al., 2023). These contrasting outcomes suggest that the ecological effects of enclosure are context-dependent and strongly mediated by the temporal dynamics of plant–soil feedbacks. In the early stages of enclosure, reduced disturbance facilitates vegetation recovery and increases community stability, whereas prolonged exclusion of disturbance may promote dominance by a few competitive species, suppress subordinate taxa, and ultimately reduce diversity. Such nonlinear responses help explain why enclosure can simultaneously be regarded as both a restoration strategy and, under certain conditions, a driver of homogenization in grassland ecosystems. Overall, explicitly incorporating plant–soil feedbacks provides a more mechanistic framework for understanding how enclosure, grazing, and mowing regulate long-term community stability (Klaus et al., 2018; Korell et al., 2024).

### 4.2 Impact of species turnover and niche overlap on the stability of grassland plant communities

Among the diversity indices examined, only Margalef richness responded significantly to land-use treatments, indicating that management effects were mainly expressed through species loss or gain rather than shifts in evenness or large-scale species turnover.

Our study revealed that, compared with enclosure and mowing, grazing significantly reduced the Margalef richness index. This result suggests that grazing may lead to a reduction in species richness within grassland ecosystems, potentially due to the direct disturbance of plant populations and the differential adaptive capacity of species under grazing pressure (He et al., 2022). In the absence of disturbance, grassland ecosystems exhibit greater stability (Cheng et al., 2016; N. He et al., 2011). Our monitoring experiments in temperate meadow steppe regions of China further demonstrated that enclosures and mowing may help maintain greater plant diversity, thereby increasing community stability. Here, reduced richness under grazing likely reflects selective species loss rather than wholesale reorganization of community composition.

In ecological studies, β diversity reflects the variability in species composition within a community (Whittaker, 1960). Although β diversity did not differ significantly among land use treatments, grazing consistently showed higher values compared with enclosure and mowing. This pattern suggests that grazing may increase compositional variability among sampling units, potentially through enhanced spatial heterogeneity, altered niche structure, and increased species turnover. However, the lack of statistically significant differences indicates that these effects were not strong or consistent enough across sites and years to generate clear treatment-level separation. Such context-dependent responses are common in heterogeneous grassland systems, where local environmental variation and interannual climatic fluctuations can obscure management effects on β diversity. Similar non-significant trends toward higher compositional variability under grazing have been reported previously, where grazing-induced species replacement may occur without leading to clear treatment-level differentiation (Ma et al., 2024).

Species turnover and niche overlap are key regulatory mechanisms linking community stability and diversity (Volf et al., 2016; Hodapp et al., 2018; Yin et al., 2021). Our study revealed that the Jaccard similarity indices were greater under the grazing and mowing treatments (**Figure 8**), indicating greater degrees of niche overlap under these management practices. When species turnover frequently occurs, community stability may be threatened by frequent species fluctuations and niche changes, whereas lower niche overlap under fenced treatments is conducive to maintaining community stability.

Furthermore, we believe that the impact of grassland management measures on species composition and turnover is reflected not only in direct disturbance or protection but also in the regulation of interspecies competition patterns through resource partitioning, thereby influencing dynamic changes in community structure. Under fenced conditions, reduced disturbance promotes stratified resource use and stable community structure, whereas grazing and mowing lower vegetation height, alleviate light competition, and accelerate species turnover (Chesson, 2000).

In addition, different management practices alter patterns of resource acquisition, which is reflected in the selection of functional traits within plant communities. Under grazing conditions, selective feeding by livestock modifies nutrient-use strategies, favoring grazing-tolerant and nutrient-efficient species and thereby accelerating the replacement of grazing-sensitive plants. Under mowing, repeated biomass removal promotes species with rapid regrowth and high reproductive capacity, enabling plants with strong regenerative traits to dominate the community (Jäkäläniemi et al., 2005). In contrast, long-term enclosure creates relatively undisturbed conditions that facilitate stratified and balanced resource use, allowing species with contrasting functional traits to coexist and thus enhancing overall community stability (Loreau et al., 2001). Together, these patterns indicate that land management regulates community stability by reshaping the balance between species turnover and niche differentiation.

### 4.3 Impact of environmental factors on plant biodiversity and community stability

The mechanisms by which environmental factors influence community diversity and stability vary under different land management practices. We separately discuss the mechanisms of influence under three land management practices—enclosure, grazing, and mowing—and predict the potential impact of future climate change on grassland ecosystems.

Under enclosure land use practices, soil nutrients (such as organic carbon, alkali-hydrolyzable nitrogen, and total nitrogen) and soil pH significantly influence plant diversity and community stability. A positive correlation was found between soil pH and community stability, with precipitation influencing soil moisture, which in turn affected soil pH; this suggests that soil acidification could have a significant effect on the long-term stability of communities (Liu et al., 2022; Token et al., 2022). Further path analysis revealed that increased precipitation may lead to excessive accumulation of soil moisture, which then influences community stability through changes in soil pH. Our results reveal the complex mechanisms by which the climate–vegetation–soil system jointly drives community stability. These results indicate that, under enclosure, plant–soil feedbacks act as a stabilizing mechanism, whereby improved soil nutrient status and moderated soil acidity enhance vegetation persistence and buffer community dynamics against climatic variability. Future climate change may exacerbate precipitation fluctuations and influence soil acidity, which could, in turn, affect species competition and community stability (Lv et al., 2023; Qiao et al., 2023).

Under grazing conditions, soil bulk density, precipitation, and soil moisture exert a significant influence on plant diversity and community stability. The positive relationship between soil bulk density and soil moisture regulates plant diversity by affecting plant growth and abundance. Furthermore, elevation and geographic location also have a significant impact on community stability under such grazing scenarios. Path analysis revealed that, by regulating soil moisture, precipitation and soil bulk density increased plant diversity and positively affected community stability. However, the negative impacts of geographic location and plant diversity on community stability suggest that certain high-altitude or low-precipitation areas may be more vulnerable to disturbances from external environmental changes (Hautier et al., 2015). These patterns suggest that grazing alters plant–soil feedbacks primarily through soil compaction, which constrains root development, modifies water availability, and weakens the stabilizing feedback between vegetation and soil processes. Notably, when soil-mediated feedbacks are weakened by grazing pressure, increases in plant diversity do not necessarily translate into higher community stability. Therefore, in the environmental management of grazing areas, it is essential to consider the synergistic effects of precipitation, soil bulk density, and geographic location. Precision grazing strategies should be developed, with stocking rates dynamically adjusted according to precipitation levels to prevent cascading collapses.

Under mow conditions, although soil moisture and precipitation significantly influence community stability, the correlations between plant diversity and environmental factors are relatively weak. Precipitation positively affected community stability by influencing soil pH, suggesting that changes in precipitation may indirectly impact long-term community stability by regulating soil acidity. Precipitation remained a key factor influencing community stability, particularly in arid and semiarid regions. This indicates that mowing generates relatively transient plant–soil feedbacks, with soil-mediated effects on community stability being strongly contingent on mowing intensity, frequency, and timing. As precipitation increases, changes in soil pH may promote the overgrowth of certain plant populations, thereby affecting community stability.

The enclosed plots can be used to simulate the natural state of grasslands undisturbed by animal and human activities. In contrast, grazing significantly affects soil compaction through herbivory, increasing the soil bulk density (Zhou et al., 2010). Under mowing conditions, human interventions such as fertilization and mowing lead to nutrient loss from the soil, thereby affecting plant diversity and community stability (Molina et al., 2021). Environmental factors, including soil nutrients, precipitation, and temperature, influence the composition of plant communities, species interactions, and functional group proportions, ultimately affecting community diversity and stability. Different land management practices impact environmental factors and community stability through distinct mechanisms. Together, these results demonstrate that plant–soil feedbacks provide a unifying framework for predicting how enclosure, grazing, and mowing differentially regulate grassland community stability.

Future climate change may lead to increased instability in terms of precipitation and temperature, further influencing soil moisture and soil physicochemical properties, which in turn affect plant community structure and functional group composition (Garcia-Palacios et al., 2018). Increased precipitation may lead to excess soil moisture, whereas higher temperatures may cause greater evaporation, thereby influencing soil acidity and nutrient cycling. In this context, future climate change could alter the species composition and functional group structure of grassland plant communities, ultimately affecting community diversity and stability. The mechanisms by which climate change impacts different land management practices need special attention, particularly in areas with high precipitation variability (Post, 2013; De Boeck et al., 2018). Our results suggest that the sensitivity of plant–soil feedbacks to climatic variability differs among management regimes, potentially amplifying or buffering climate impacts on community stability.

The traditional diversity–stability hypotheses posit that greater species diversity enhances ecosystem stability through insurance and overyielding effects. In contrast, resilience theory emphasizes an ecosystem’s capacity to absorb disturbances and recover its structure and function following perturbations, that is, its resilience (Holling, 1973; Pimm, 1984). These two frameworks differ in explaining ecosystem responses to perturbations: while the diversity–stability hypothesis focuses on functional complementarity and redundancy among species, resilience theory emphasizes recovery dynamics and system reorganization. In our study, we observed that under the grazing and mowing treatments, despite greater species diversity, community stability did not correspondingly improve and, in some cases, even declined. This observation challenges the assumption that high species turnover (i.e., high diversity) necessarily leads to increased stability. Our results indicate that without supportive plant–soil feedbacks, increased diversity alone is insufficient to maintain ecosystem stability.

Based on these observations, we propose a “management-mediated turnover–niche trade-off model.” This model posits that land management practices influence community stability by modulating species turnover and niche differentiation. Here, we further emphasize that plant–soil feedbacks regulate the strength and direction of this trade-off by mediating resource availability, soil physical constraints, and vegetation recovery dynamics. Specifically, enclosure treatment may reduce external disturbances, thereby lowering species turnover and promoting niche differentiation, which in turn enhances community stability. Conversely, grazing and mowing may increase species turnover, leading to greater niche overlap, intensified resource competition, and ultimately reduced community stability. This model integrates elements of both the diversity–stability hypothesis and resilience theory, underscoring the critical role of land management in regulating community stability. Future research should further validate this model and explore the mechanisms by which management practices affect community stability across different ecosystems.

## 5 Conclusions

Our study demonstrated that grassland management profoundly shapes the structure and stability of grassland ecosystems in the Hulunbuir temperate meadow steppe. By minimizing disturbances, closure fosters greater species richness and enhanced community resilience, as evidenced by more stable biomass production. In contrast, grazing reduces diversity and stability through intensified herbivory and trampling, which accelerate species turnover and increase soil compaction. Mowing exerts intermediate effects, subtly altering competitive interactions and species composition. Critically, our findings highlight that the interplay between species turnover and niche overlap is a key mechanism underpinning community stability. Environmental factors—especially soil bulk density, nitrogen, and organic carbon—further modulate these dynamics by influencing resource availability and interspecific competition. Structural equation modelling confirmed that both the direct effects of these environmental drivers and their indirect effects via alterations in species interactions are essential for maintaining ecosystem function. Our results underscore the need for context-specific management strategies that balance disturbance regimes to conserve biodiversity and sustain ecosystem services. Future work should extend these observations over longer time scales and under variable climatic conditions to refine management practices for temperate grasslands globally.

## Data Availability

The datasets presented in this article are not readily available because Proprietary data were used in this study. Requests to access the datasets should be directed to yanruirui@caas.cn.
