# Supplementary material for: Enclosure enhances grassland plant diversity and community stability more effectively than grazing and mowing by strengthening species turnover regulation and soil process driving
Source: Front Plant Sci. 2026 Feb 18;17:1747083. doi: 10.3389/fpls.2026.1747083 (PMC12956662; doi:10.3389/fpls.2026.1747083)
Supplement: Supplementary file 1 [file DataSheet1.pdf]

Supplementary Table

Table S1. Results of one-way analysis of variance (ANOVA) for plant height among different land-use treatments across three consecutive years (2022 – 2024).

Year	Source of variation	Sum of squares	df	Mean square	F value	p value
2022	Between groups	2,090.742	2	1,045.371	14.703	0.001
	Within groups	782.100	11	71.100	—	—
	Total	2,872.842	13	—	—	—
2023	Between groups	613.627	2	306.813	15.833	0.001
	Within groups	213.162	11	19.378	—	—
	Total	826.789	13	—	—	—
2024	Between groups	683.452	2	341.726	9.561	0.004
	Within groups	393.163	11	35.742	—	—
	Total	1,076.615	13	—	—	—

Values represent the results of one-way ANOVA testing the effects of land-use treatments on plant height for each year separately. df, degrees of freedom. Significant differences were determined at $p < 0.05$.

Table S2. Results of one-way analysis of variance (ANOVA) for plant height among different land-use treatments based on pooled data from 2022 to 2024.

Source of variation	Sum of squares	df	Mean square	F value	p value
Between groups	2973.994	2	1486.997	13.812	< 0.001
Within groups	4198.660	39	107.658	—	—
Total	7172.653	41	—	—	—

Values represent the results of one-way ANOVA testing the effects of land-use treatments on plant height using pooled data across the three study years (2022 – 2024). df, degrees of freedom. Significant differences were determined at $p < 0.05$.

Table S3. Results of one-way analysis of variance (ANOVA) for plant cover among different land-use treatments across three consecutive years (2022 – 2024).

Year	Source of variation	Sum of squares	df	Mean square	F value	p value
2022	Between groups	91.295	2	45.647	0.553	0.590

	**Within groups**	907.856	11	82.532	—	—
	**Total**	999.151	13	—	—	—
**2023**	**Between groups**	96.756	2	48.378	0.237	0.793
	**Within groups**	2,246.385	11	204.217	—	—
	**Total**	2,343.141	13	—	—	—
**2024**	**Between groups**	1,947.113	2	973.556	5.475	0.022
	**Within groups**	1,956.017	11	177.820	—	—
	**Total**	3,903.130	13	—	—	—

Values represent the results of one-way ANOVA testing the effects of land-use treatments on plant cover for each year separately. df, degrees of freedom. Significant differences were determined at $p < 0.05$.

Table S4. Results of one-way analysis of variance (ANOVA) for plant cover among different land-use treatments based on pooled data from 2022 to 2024.

Source of variation	Sum of squares	df	Mean square	F value	p value
**Between groups**	1267.462	2	633.731	4.095	0.024
**Within groups**	6035.942	39	154.768	—	—
**Total**	7303.404	41	—	—	—

Values represent the results of one-way ANOVA testing the effects of land-use treatments on plant cover using pooled data across the three study years (2022 – 2024). df, degrees of freedom. Significant differences were determined at $p < 0.05$.

Table S5. Results of one-way analysis of variance (ANOVA) for plant density among different land-use treatments across three consecutive years (2022 – 2024).

Year	Source of variation	Sum of squares	df	Mean square	F value	p value
**2022**	**Between groups**	177,003.127	2	88,501.563	2.173	0.160
	**Within groups**	447,948.995	11	40,722.636	—	—
	**Total**	624,952.122	13	—	—	—
**2023**	**Between groups**	555,192.655	2	277,596.327	3.861	0.054
	**Within groups**	790,898.274	11	71,899.843	—	—
	**Total**	1,346,090.929	13	—	—	—

**2024**	**Between groups**	557,685.071	2	278,842.536	0.748	0.496
	**Within groups**	4,100,662.429	11	372,787.494	—	—
	**Total**	4,658,347.500	13	—	—	—

Values represent the results of one-way ANOVA testing the effects of land-use treatments on plant density for each year separately. df, degrees of freedom. Significant differences were determined at $p < 0.05$.

Table S6. Results of one-way analysis of variance (ANOVA) for plant density among different land-use treatments based on pooled data from 2022 to 2024.

Source of variation	Sum of squares	df	Mean square	F value	p value
**Between groups**	1,029,911.964	2	514,955.982	3.496	0.040
**Within groups**	5,743,939.378	39	147,280.497	—	—
**Total**	6,773,851.342	41	—	—	—

Values represent the results of one-way ANOVA testing the effects of land-use treatments on plant density using pooled data across the three study years (2022 – 2024). df, degrees of freedom. Significant differences were determined at $p < 0.05$.

Table S7. Results of one-way analysis of variance (ANOVA) for aboveground biomass among different land-use treatments across three consecutive years (2022–2024).

Year	Source of variation	Sum of squares	df	Mean square	F value	p value
**2022**	**Between groups**	45,566.122	2	22,783.061	3.434	0.069
	**Within groups**	72,972.627	11	6,633.875	—	—
	**Total**	118,538.749	13	—	—	—
**2023**	**Between groups**	32,988.713	2	16,494.357	4.437	0.039
	**Within groups**	40,896.609	11	3,717.874	—	—
	**Total**	73,885.323	13	—	—	—
**2024**	**Between groups**	39,408.832	2	19,704.416	16.059	0.001
	**Within groups**	13,497.360	11	1,227.033	—	—
	**Total**	52,906.192	13	—	—	—

Values represent the results of one-way ANOVA testing the effects of land-use treatments on aboveground biomass for each year separately. df, degrees of freedom. Significant differences were determined at $p < 0.05$.

Table S8. Results of one-way analysis of variance (ANOVA) for aboveground biomass among different land-use treatments based on pooled data from 2022 to 2024.

Source of variation	Sum of squares	df	Mean square	F value	p value
Between groups	110,415.769	2	55,207.885	13.329	< 0.001
Within groups	161,541.337	39	4,142.086	—	—
Total	271,957.106	41	—	—	—

Values represent the results of one-way ANOVA testing the effects of land-use treatments on aboveground biomass using pooled data across the three study years (2022 – 2024). df, degrees of freedom. Significant differences were determined at $p < 0.05$.

Table S9. Results of one-way ANOVA for importance values of major plant families under different land-use treatments (pooled data from 2022 – 2024)

Plant family	Source of variation	Sum of squares	df	Mean square	F value	p value
Asteraceae	Between groups	246.398	2	123.199	2.467	0.098
	Within groups	1,947.240	39	49.929	—	—
	Total	2,193.637	41	—	—	—
Poaceae	Between groups	270.019	2	135.010	1.024	0.369
	Within groups	5,144.053	39	131.899	—	—
	Total	5,414.072	41	—	—	—
Cyperaceae	Between groups	215.175	2	107.588	1.420	0.254
	Within groups	2,955.153	39	75.773	—	—
	Total	3,170.328	41	—	—	—
Fabaceae	Between groups	172.373	2	86.186	7.649	0.002
	Within groups	439.411	39	11.267	—	—
	Total	611.784	41	—	—	—
Ranunculaceae	Between groups	202.834	2	101.417	5.585	0.007
	Within groups	708.161	39	18.158	—	—
	Total	910.996	41	—	—	—
Rosaceae	Between	288.532	2	144.266	5.272	0.009

	**groups**					
	**Within groups**	1,067.252	39	27.365	—	—
	**Total**	1,355.784	41	—	—	—
**Plantaginaceae**	**Between groups**	510.319	2	255.159	9.149	0.001
	**Within groups**	1,087.659	39	27.889	—	—
	**Total**	1,597.978	41	—	—	—
**Other families**	**Between groups**	134.549	2	67.275	1.319	0.279
	**Within groups**	1,988.597	39	50.990	—	—
	**Total**	2,123.146	41	—	—	—

Values represent the results of one-way ANOVA testing the effects of land-use treatments on the importance values of major plant families using pooled data from 2022 to 2024. df, degrees of freedom. Significant differences were determined at $p < 0.05$.

Table S10. Results of one-way ANOVA for relative aboveground biomass of major plant families under different land-use treatments (pooled data from 2022 – 2024)

Plant family	Source of variation	Sum of squares	df	Mean square	F value	p value
**Asteraceae**	**Between groups**	178.873	2	89.436	0.414	0.664
	**Within groups**	8,428.100	39	216.105	—	—
	**Total**	8,606.973	41	—	—	—
**Poaceae**	**Between groups**	1,388.764	2	694.382	2.041	0.144
	**Within groups**	13,269.782	39	340.251	—	—
	**Total**	14,658.547	41	—	—	—
**Cyperaceae**	**Between groups**	261.514	2	130.757	1.656	0.204
	**Within groups**	3,078.716	39	78.941	—	—
	**Total**	3,340.230	41	—	—	—
**Fabaceae**	**Between groups**	217.639	2	108.819	4.416	0.019
	**Within groups**	961.109	39	24.644	—	—
	**Total**	1,178.747	41	—	—	—
**Ranunculaceae**	**Between**	300.563	2	150.282	3.895	0.029

	groups					
	Within groups	1,504.642	39	38.581	—	—
	Total	1,805.205	41	—	—	—
Rosaceae	Between groups	899.267	2	449.634	5.808	0.006
	Within groups	3,019.380	39	77.420	—	—
	Total	3,918.647	41	—	—	—
Plantaginaceae	Between groups	1,038.468	2	519.234	8.765	0.001
	Within groups	2,310.398	39	59.241	—	—
	Total	3,348.866	41	—	—	—
Other families	Between groups	66.850	2	33.425	0.624	0.541
	Within groups	2,089.530	39	53.578	—	—
	Total	2,156.381	41	—	—	—

Values represent the results of one-way ANOVA testing the effects of land-use treatments on the relative aboveground biomass of major plant families using pooled data from 2022 to 2024. df, degrees of freedom. Significant differences were determined at $p < 0.05$.

Table S11. Results of one-way ANOVA for the Shannon–Wiener diversity index under different land-use treatments from 2022 to 2024.

Year	Source of variation	Sum of squares	df	Mean square	F value	p value
2022	Between groups	0.195	2	0.097	0.412	0.672
	Within groups	2.599	11	0.236	—	—
	Total	2.794	13	—	—	—
2023	Between groups	0.624	2	0.312	2.861	0.100
	Within groups	1.200	11	0.109	—	—
	Total	1.824	13	—	—	—
2024	Between groups	0.265	2	0.133	0.770	0.487
	Within groups	1.896	11	0.172	—	—
	Total	2.161	13	—	—	—

Values represent the results of one-way ANOVA testing the effects of land-use treatments on the Shannon–Wiener diversity index for each year separately. df, degrees of freedom. Significant

differences were determined at $p < 0.05$.

Table S12. Results of one-way ANOVA for the Simpson diversity index under different land-use treatments from 2022 to 2024.

Year	Source of variation	Sum of squares	df	Mean square	F value	p value
2022	Between groups	0.008	2	0.004	0.478	0.632
	Within groups	0.094	11	0.009	—	—
	Total	0.102	13	—	—	—
2023	Between groups	0.010	2	0.005	2.276	0.149
	Within groups	0.024	11	0.002	—	—
	Total	0.034	13	—	—	—
2024	Between groups	0.003	2	0.002	0.514	0.612
	Within groups	0.033	11	0.003	—	—
	Total	0.036	13	—	—	—

Values represent the results of one-way ANOVA testing the effects of land-use treatments on the Simpson diversity index for each year separately. df, degrees of freedom. Significant differences were determined at $p < 0.05$.

Table S13. Results of one-way ANOVA for the Pielou evenness index under different land-use treatments from 2022 to 2024.

Year	Source of variation	Sum of squares	df	Mean square	F value	p value
2022	Between groups	0.002	2	0.001	0.129	0.880
	Within groups	0.103	11	0.009	—	—
	Total	0.106	13	—	—	—
2023	Between groups	0.008	2	0.004	2.755	0.107
	Within groups	0.016	11	0.001	—	—
	Total	0.024	13	—	—	—
2024	Between groups	0.002	2	0.001	0.177	0.840
	Within groups	0.049	11	0.004	—	—
	Total	0.051	13	—	—	—

Values represent the results of one-way ANOVA testing the effects of land-use treatments on the Pielou evenness index for each year separately. df, degrees of freedom. Significant differences were determined at $p < 0.05$.

Table S14. Results of one-way ANOVA for the Margalef richness index under different land-use treatments from 2022 to 2024.

Year	Source of variation	Sum of squares	df	Mean square	F value	p value
2022	Between groups	8.695	2	4.348	0.625	0.553
	Within groups	76.490	11	6.954	—	—
	Total	85.186	13	—	—	—
2023	Between groups	31.216	2	15.608	4.305	0.042
	Within groups	39.885	11	3.626	—	—
	Total	71.101	13	—	—	—
2024	Between groups	21.370	2	10.685	4.149	0.045
	Within groups	28.331	11	2.576	—	—
	Total	49.701	13	—	—	—

Values represent the results of one-way ANOVA testing the effects of land-use treatments on the Margalef richness index for each year separately. df, degrees of freedom. Significant differences were determined at $p < 0.05$.

Table S15. Results of one-way ANOVA for the Whittaker beta-diversity index under different land-use treatments from 2022 to 2024.

Year	Source of variation	Sum of squares	df	Mean square	F value	p value
2022	Between groups	0.249	2	0.124	0.134	0.876
	Within groups	10.214	11	0.929	—	—
	Total	10.463	13	—	—	—
2023	Between groups	1.930	2	0.965	0.649	0.541
	Within groups	16.346	11	1.486	—	—
	Total	18.276	13	—	—	—
2024	Between groups	5.596	2	2.798	1.613	0.243

	**Within groups**	19.080	11	1.735	—	—
	**Total**	24.676	13	—	—	—

Values represent the results of one-way ANOVA testing the effects of land-use treatments on the Whittaker beta-diversity index for each year separately. df, degrees of freedom. Significant differences were determined at $p < 0.05$.

Table S16. Results of one-way ANOVA for the gamma-diversity index under different land-use treatments from 2022 to 2024.

Year	Source of variation	Sum of squares	df	Mean square	F value	p value
**2022**	**Between groups**	26.441	2	13.220	0.660	0.536
	**Within groups**	220.314	11	20.029	—	—
	**Total**	246.755	13	—	—	—
**2023**	**Between groups**	44.341	2	22.170	0.861	0.450
	**Within groups**	283.396	11	25.763	—	—
	**Total**	327.737	13	—	—	—
**2024**	**Between groups**	9.618	2	4.809	0.145	0.867
	**Within groups**	365.115	11	33.192	—	—
	**Total**	374.733	13	—	—	—

Values represent the results of one-way ANOVA testing the effects of land-use treatments on the gamma-diversity index for each year separately. df, degrees of freedom. Significant differences were determined at $p < 0.05$.

Table S17. Results of one-way ANOVA for species richness among different land-use treatments from 2022 to 2024.

Year	Source of variation	Sum of squares	df	Mean square	F value	p value
**2022**	**Between groups**	1,048.012	2	524.006	11.417	0.002
	**Within groups**	504.845	11	45.895	—	—
	**Total**	1,552.857	13	—	—	—
**2023**	**Between groups**	1,406.214	2	703.107	20.089	< 0.001
	**Within groups**	385.000	11	35.000	—	—
	**Total**	1,791.214	13	—	—	—

**2024**	**Between groups**	1,070.655	2	535.327	5.802	0.019
	**Within groups**	1,014.845	11	92.259	—	—
	**Total**	2,085.500	13	—	—	—

Values represent the results of one-way ANOVA testing the effects of land-use treatments on species richness for each year separately. df, degrees of freedom. Significant differences were determined at $p < 0.05$.

Table S18. Results of one-way ANOVA for soil physicochemical properties and nutrient contents under different land-use treatments (pooled data from 2022 – 2024).

**Soil variable**	**Source of variation**	**Sum of squares**	**df**	**Mean square**	**F value**	**p value**
**SBD**	**Between groups**	0.192	2	0.096	3.967	0.027
	**Within groups**	0.943	39	0.024	—	—
	**Total**	1.135	41	—	—	—
**pH**	**Between groups**	0.807	2	0.404	5.402	0.008
	**Within groups**	2.914	39	0.075	—	—
	**Total**	3.722	41	—	—	—
**SM**	**Between groups**	242.606	2	121.303	2.708	0.079
	**Within groups**	1,747.081	39	44.797	—	—
	**Total**	1,989.687	41	—	—	—
**OC**	**Between groups**	994.615	2	497.307	7.366	0.002
	**Within groups**	2,633.101	39	67.515	—	—
	**Total**	3,627.716	41	—	—	—
**AN**	**Between groups**	21,400.070	2	10,700.035	5.188	0.010
	**Within groups**	80,433.144	39	2,062.388	—	—
	**Total**	101,833.214	41	—	—	—
**TN**	**Between groups**	7.393	2	3.696	7.797	0.001
	**Within groups**	18.489	39	0.474	—	—
	**Total**	25.882	41	—	—	—

**TP**	**Between groups**	0.091	2	0.045	3.136	0.055
	**Within groups**	0.564	39	0.014	—	—
	**Total**	0.654	41	—	—	—
**TK**	**Between groups**	5.848	2	2.924	0.220	0.804
	**Within groups**	518.290	39	13.289	—	—
	**Total**	524.138	41	—	—	—
**AP**	**Between groups**	1.404	2	0.702	0.300	0.742
	**Within groups**	91.220	39	2.339	—	—
	**Total**	92.625	41	—	—	—
**AK**	**Between groups**	34,587.707	2	17,293.854	4.117	0.024
	**Within groups**	163,805.225	39	4,200.134	—	—
	**Total**	198,392.932	41	—	—	—

Values represent the results of one-way ANOVA testing the effects of land-use treatments on soil physicochemical properties and nutrient contents using pooled data from 2022 to 2024. df, degrees of freedom. Significant differences were determined at $p < 0.05$.
